# Total Antioxidant Capacity in HBV Carriers, a Promising Biomarker for Evaluating Hepatic Fibrosis: A Pilot Study

**DOI:** 10.3390/antiox10010077

**Published:** 2021-01-08

**Authors:** Jing-Hua Wang, Sung-Bae Lee, Dong-Soo Lee, Chang-Gue Son

**Affiliations:** 1 Institute of Bioscience & Integrative Medicine, Daejeon University, 75, Daedeok-daero 176, Seo-gu, Daejeon 35235, Korea; ewccwang@gmail.com (J.-H.W.); sky161300@naver.com (S.-B.L.); 2 Department of Internal Medicine, Daejeon St. Mary’s Hospital, College of Medicine, The Catholic University of Korea, 64, Daeheung-ro, Jung-gu, Daejeon 34943, Korea; endoscope@hanmail.net

**Keywords:** hepatitis B virus, oxidative stress, total antioxidant capacity, liver fibrosis, liver stiffness measurement, AST to platelet index

## Abstract

Oxidative stress plays a pivotal role in the progression of chronic hepatitis B; however, it is unclear whether the status of blood oxidative stress and antioxidant components differs depending on the degree of hepatic fibrosis. To explore the relationship between oxidative stress/antioxidant capacity and the extent of hepatic fibrosis, fifty-four subjects with liver fibrosis (5.5 ≤ liver stiffness measurement (LSM) score ≤ 16.0 kPa) by chronic hepatitis B virus (HBV) were analyzed. From the analysis of eight kinds of serum oxidative stress/antioxidant profiles and liver fibrosis degrees, the level of total antioxidant capacity (TAC) reflected a negative correlation with the severity of hepatic fibrosis (Pearson correlation, r = −0.35, *p* = 0.01). Moreover, TAC showed higher sensitivity (73.91%) than the aspartate transaminase (AST) to platelet ratio index (APRI, 56.52%) in the receiver operating characteristic (ROC) curves. Interestingly, the TAC level finely reflected the fibrosis degree in inactive carriers (HBV DNA < 2000 IU/mL), while the APRI did in active carriers (HBV DNA > 2000 IU/mL). In conclusion, TAC is a promising biomarker for evaluating the progression of liver fibrosis in patients with HBV, and this finding may indicate the involvement of TAC-composing factors in the pathogenesis of hepatic fibrosis in chronic HBV carriers.

## 1. Introduction

Generally, chronic liver disease can be induced by a variety of causes, including hepatitis B virus (HBV) infection [1]. Meanwhile, it has been conservatively estimated that two billion people have been infected with HBV, and that at least 400 million have become HBV carriers worldwide [2]. In highly endemic areas, HBV has been a predominant cause of chronic liver diseases, including hepatic fibrosis, cirrhosis, and cancer. Although global vaccination has rapidly lowered the HBV infection rate worldwide [3], chronic hepatitis B is still a considerable public health issue, as the seventh highest cause of death worldwide, especially in South Korea and China [3,4]. In addition, the progression of hepatic fibrosis is a critical pathologic step determining the clinical outcome in subjects with chronic liver disease, including HBV infection [5,6]. Chronic HBV infection is a typical cause of liver fibrogenesis. Previous clinical studies reported that 20% to 30% of HBV carriers with normal alanine transaminase (ALT) levels and high HBV DNA load had hepatic fibrosis [7].

Regarding the hepatic fibrosis-related pathologic process, oxidative stress is mostly adapted as a common contributor [8,9]. Oxidative stress is a ubiquitous phenomenon that exists in most living organisms and is characterized by a loss of equilibrium between free radicals such as reactive oxygen species (ROS) and antioxidant capacity [10,11]. Although moderate oxidative stress exerts an essential role in physiological processes, such as intracellular signal transduction and a natural defense against pathogenic microorganisms [12,13], supraphysiological oxidative stress is undoubtedly implicated in the initiation or progression of numerous disorders, including liver fibrosis [11]. Therefore, antioxidative therapy is frequently used for chronic hepatic disorders and has been used to protect against liver fibrosis in many clinical trials [14,15].

In the clinical field of hepatic fibrosis, in addition to the development of antifibrotic agents, the rapid and accurate assessment of the stage of liver fibrosis is a very important issue [16]. To date, liver biopsy remains to be the gold standard for the diagnosis of liver fibrosis; however, invasive operation, sampling error, and cost seriously limit its widespread and frequent use in clinics. Fortunately, a novel noninvasive tool, namely, liver stiffness measurement (LSM) by transient elastography technology (e.g., FibroScan, Echosens, Paris, France), has been developed to estimate liver scarring with high accuracy [17]. In addition, noninvasive biomarkers have been commonly utilized, such as the aspartate transaminase (AST) to platelet ratio index (APRI), fibrosis-4 (FIB-4), and hyaluronic acid (HA), which are created based on the pathophysiological features of hepatic fibrosis [18,19]. Among those, the APRI, which reflects the decrease in blood platelet number and increase in serum levels of AST in hepato-fibrotic changes, has been mostly preferred for detecting liver fibrosis/cirrhosis [20].

Numerous studies have indicated that oxidative stress is excessive in patients with chronic HBV hepatitis as compared with healthy subjects [21], but the comprehensive profiling of oxidative stress and antioxidative status within HBV carriers according to liver fibrosis degree is still unclear. It is worth noting whether oxidative stress/antioxidant-related parameters can be used as reliable predictive factors for assessing liver fibrosis in HBV carriers. Therefore, we analyzed the serum oxidative/antioxidative profiles comparing hepatofibrotic severity among chronic HBV carriers and verified the correlations in mild to moderate/severe levels of liver fibrosis.

## 2. Subjects and Methods

### 2.1. Subjects and Study Design

Ethical approval for the study was obtained from the institutional ethics committee of the Daejeon University Dunsan Hospital (DJMC200901) and the Daejeon St. Mary’s Hospital of Catholic University of Korea (DC12MDMT0041), and all methods were performed according to the relevant guidelines and regulations of the institution. Voluntary written informed consent was obtained from all participants. HBV virus-infected participants (aged 18–65 years) with disorders with a 5.5 ≤ LSM score ≤ 16.0 kPa using FibroScan were recruited (Table 1). However, participants with marked liver cirrhosis (>16.0 kPa of LSM) who took immunosuppressive, antioxidative, and anti-inflammatory drugs; cytotoxic drugs; or hormone therapy were excluded from this study. Detailed inclusion/exclusion criteria are shown in the Supplementary Materials (Table S1). Blood samples were collected from all subjects after an overnight fast of at least 8 h. The serum was separated under reactive centrifugal force (RCM) centrifugation at 3000× *g* for 15 min at 4 °C and eventually stored at −80 °C for future determination.

### 2.2. Liver Stiffness Measurement by FibroScan

FibroScan (Echosence, Paris, France), a noninvasive liver fibrosis diagnostic tool using transient elastography technology, was used in this study to evaluate the extent of liver fibrosis. All FibroScan tests were performed after overnight fasting. To compare the oxidative/antioxidant parameters in accordance with the level of hepatofibrosis, we divided subjects into two groups by LSM score, i.e., from 8.5 kPa to 16.0 kPa versus from 5.5 kPa to 8.5 kPa, on the basis of a previous study [22].

### 2.3. Complete Blood Count, Fasting Blood Glucose, and Serum Biochemical Analysis

The complete blood count (CBC) was determined using an automated hematology analyzer (Sysmex XE-2100, Kobe, Japan). The fasting blood glucose (FBG) levels were checked by Accu-Chek Glucometer (Roche Diabetes Care GmbH, Mannheim, Germany). The serum levels of alkaline aspartate transaminase (AST), alanine transaminase (ALT), alanine phosphatase (ALP), total protein, albumin, uric acid (UA), bilirubin, total cholesterol, high-density lipoprotein (HDL), and triglyceride (TG) were measured by an autochemistry analyzer (Chiron, Emeryville, CA, USA). The levels of serum hyaluronic acid (HA), transforming growth factor beta (TGF-β), and platelet-derived growth factor (PDGF) were analyzed by commercial ELISA kits (R&D Systems, Minneapolis, MN, USA).

### 2.4. Serum Lipid Peroxide Determination

The serum lipid peroxide levels were measured using thiobarbituric acid reactive substances (TBARS), according to the modified Satoh’s method [23]. Briefly, the TBARS concentration was expressed as μM malondialdehyde (MDA) in serum. One hundred microliters of undiluted serum or various concentrations of 1,1,3,3-tetraethoxypropane (TEP) as a standard was added to 500 μL of 0.02% trichloroacetic acid (TCA) for a 10 min incubation, mixed with 500 μL of sulfuric acid and 600 μL of 20 mg/dL thiobarbituric acid (TBA) in 2 M sodium sulfate, heated at 100 °C for 60 min, cooled on ice for 10 min, centrifuged to remove the supernatant, and vigorously vortexed with 300 μL of n-butanol. After centrifugation at 3000× *g* for 10 min, the absorbance of the upper organic layer was detected at 530 nm with a spectrophotometer (Molecular Devices, Sunnyvale, CA, USA) and was compared with the TEP standard curve.

### 2.5. Serum Total Reactive Oxygen Species Determination

Total reactive oxygen species (ROS) is a primary cause of oxidative stress [24]. In order to assess the whole status of oxidative stress in vivo, the serum ROS level was assayed by using Hayashi’s method [25]. In brief, 5 μL of undiluted serum or different concentration of hydrogen peroxide (H_2_O_2_) standard solution was mixed with 140 μL of 0.1 M sodium acetate buffer (pH 4.8) in a 96-well plate at room temperature, and then the mixture was incubated at 37 °C for 5 min. The 100 μL of 10 mM N, N-diethyl-para-phenylenediamine (DEPPD) and 4.37 μM ferrous sulfate mixture (1:25, *v*/*v*) was added to each well, and after preincubation at 37 °C for 1 min, the level of serum ROS was determined at 505 nm using a spectrophotometer (Molecular Devices).

### 2.6. Serum Superoxide Dismutase Determination

The serum superoxide dismutase (SOD) activity was determined spectrophotometrically using a commercial SOD assay kit (Dojindo Laboratories, Kumamoto, Japan). One unit of SOD activity was defined as the quantity of enzyme inhibiting the reduction reaction of highly water-soluble tetrazolium salt, namely WST-1 (2-(4-Iodophenyl)-3-(4-nitrophenyl)-5-(2,4-disulfophenyl)-2H-tetrazolium, monosodium salt), with superoxide anion. According to the manufacturer’s technical manual, absorbance was measured at 450 nm using a spectrophotometer (Molecular Devices). A series dilution of lyophilized bovine erythrocyte SOD was used to make a standard curve ranging from 0 to 50 U/mL.

### 2.7. Serum Catalase Determination

The serum catalase peroxidatic activity was evaluated by Wheeler’s method [26]. Briefly, phosphatase buffer (30 μL, 250 mM, pH 7.0), methanol (30 μL, 12 mM) and H_2_O_2_ (30 μL, 44 mM) were mixed together with diluted serum samples or catalase standard solutions in 96-well plates. After 10 min of incubation at RT, the reaction was stopped by adding Purpald solution (90 μL, 22.8 mM Purpald in 2 N potassium hydroxide). After adding the potassium peroxide solution (30 μL, 65.2 mM in 0.5 N potassium hydrate), the mixture was incubated for 10 min at RT. The absorbance of the purple formaldehyde adduct was measured at 550 nm using a spectrophotometer (Molecular Devices)

### 2.8. Serum Total Glutathione Content, Glutathione Peroxidase, and Glutathione Reductase Activity Determination

The serum glutathione (GSH) content was assessed by an EZ-Glutathione Assay Kit (DoGen Bio Co., Ltd., Seoul, Korea). Glutathione peroxidase (GPx) and glutathione reductase (GRed) were determined using the glutathione peroxidase cellular activity assay kit and glutathione reductase assay kit, respectively (Sigma-Aldrich, St. Louis, MO, USA). The above assays were performed strictly following the manufacturer’s instructions.

### 2.9. Serum Total Antioxidant Capacity Assay

The serum total antioxidant capacity (TAC) was evaluated using Kambsyashi’s method [27]. Briefly, 90 μL of 10 mM phosphate-buffered saline (PBS, pH 7.2), 50 μL of 18 μM myoglobin solution and 20 μL of 3 mM 2,2′-azino-bis (3-ethylbenzthiazoline-6-sulfonic acid) diammonium salt (ABTS) solution were mixed simultaneously with two times diluted serum samples or various concentrations of standard gallic acid solution for 3 min at room temperature (RT). Then, the reaction was started by adding 20 μL of 30% H_2_O_2_ and incubating for 5 min at RT. The absorbance measurements were performed by using a microplate reader (Molecular Device, CA, USA) at 600 nm. The TAC was expressed as the gallic acid equivalent antioxidant capacity (GEAC).

### 2.10. Statistical Analysis

All results are expressed as the mean ± standard deviation. The statistical package for science software (SPSS, 17.0 version, Chicago, IL, USA) was used for statistical analyses. The statistical significance of differences was analyzed using Student’s *t*-test. The relationship strength among parameters was assessed by the two-tailed Pearson’s correlation test. A value of Pearson’s correlation coefficient r > 0.3 or <−0.3 was considered to be a significant positive or negative correlation, respectively. To determine the diagnostic utility of related parameters, receiver operating characteristic (ROC) curves and the area under the curve (AUC) were analyzed by MedCalc Statistical software (version 19.1); *p* < 0.05 was considered to indicate a significant difference.

## 3. Results and Discussion

Chronic HBV infection is deemed to be one of the major causes of liver fibrogenesis [28,29]. Although multiple studies have shown that liver fibrosis can be mediated by oxidative stress [30,31], it is still unclear whether serum redox biomarkers can be used to evaluate or predict the development of liver fibrosis and subsequent cirrhosis in HBV carriers. To answer this question, from two hospitals, we enrolled 54 subjects (44 males and 10 females) with liver fibrosis caused by chronic HBV. The median age and mean body mass index (BMI) were 52 years (males 51 and females 54) and 23.8 (males 24.0 and females 22.8), respectively. In this study, the inclusive range for liver fibrosis was set to 5.5 ≤ LSM score ≤ 16 kPa, and the average LSM was 8.4 kPa (males 8.6 and females 7.7) (Table 1). Even if the LSM score varies with the causative diseases, this LSM range is generally considered to conform to the 2 ≤ F ≤ 3 METAVIR score (a commonly used tool for assessing the severity of hepatic fibrosis) (F0, no fibrosis; F1, portal fibrosis; F2, portal fibrosis with few septa; F3, septal fibrosis; F4, cirrhosis on histologic examination) [32]. In a previous study of 530 adults, the mean LSM of healthy subjects was 4.1 kPa, while it was 3.4 kPa in the elderly (>55 years) group [33].

Serum TAC (A) and the APRI (D) were compared according to the LSM score (LSM > 8.5 or <8.5). The serum TAC values (B) were negatively correlated with the LSM scores in HBV carriers, and the APRI (E) was positively correlated with the LSM scores in HBV carriers. Receiver operating characteristic (ROC) curves were employed for the prediction of moderate/severe liver fibrosis (LSM > 8.5) using (C) TAC and (F) the APRI. LSM scores were compared according to the values of TAC (G) and the APRI (H) based on halves or quartiles, respectively (from low to high value).

**Figure 1:**
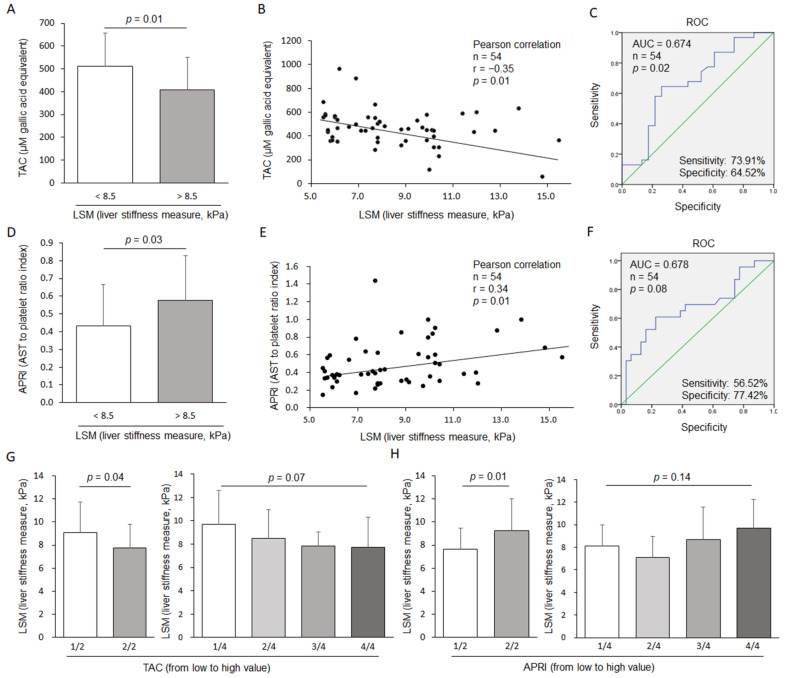
Serum total antioxidant capacity (TAC) and aspartate transaminase (AST) to platelet ratio index (APRI) for predicting moderate/severe liver fibrosis within HBV carriers. Serum TAC (**A**) and the APRI (**D**) were compared according to the LSM score (LSM > 8.5 or < 8.5). Serum TAC values (**B**) were negatively correlated with LSM scores in HBV carriers, and the APRI (**E**) was positively correlated with LSM scores in HBV carriers. Receiver operating characteristic (ROC) curves were employed for the prediction of moderate/severe liver fibrosis (LSM > 8.5) using (**C**) TAC and (**F**) the APRI. LSM scores were compared according to the values of TAC (**G**) and the APRI (**H**) based on halves or quartiles, respectively (from low to high value).

To explore the features of oxidative/antioxidant parameters by the level of hepatofibrosis, we divided subjects into two groups, i.e., 5.5 kPa ≤ LSM ≤ 8.5 kPa versus 8.5 kPa < LSM ≤ 16.0 kPa. A previous clinical study had reported that 8.5 kPa LSM was a cutoff score for bridging fibrosis in 900 chronic viral hepatitis subjects [22]. Then, we compared oxidative stress-related parameters in serum, such as ROS, MDA, TAC, SOD, catalase, GSH, GPx, and GRd. Among these, four antioxidant parameters (TAC, catalase, GSH, and GPx) showed a tendency of reduction in the LSM > 8.5 kPa group, while two oxidative parameters (ROS and MDA) did not reveal any change. Interestingly, TAC showed a significant difference according to the LSM classification (*p* = 0.01, Figure 1A and Table 2). When we performed Pearson correlation analysis, we also found a significant correlation between TAC levels and LSM scores in all 54 subjects (r = −0.35, *p* = 0.01, Figure 1B). When we compared those oxidative parameters with known hepatofibrotic markers, including the APRI, FIB-4, and HA, the APRI (but not FIB-4 and HA) also revealed differences according to the 2-grouped LSM scores (*p* = 0.03, Figure 1D and Table 2) and showed a significant correlation with the LSM score (r = 0.34, *p* = 0.01, Figure 1E). As expected, the LSM scores (hepatofibrosis) were distinguished in the reverse setting using the median values of TAC or APRI (*p* = 0.04 for TAC, *p* = 0.01 for APRI, Figure 1G,H). In particular, the TAC levels gradually decreased depending on the increase in LSM scores among the four groups, as further consistently compared to the APRI (Figure 1G,H).

The levels of TAC (A), APRI (B), FIB-4 (C), and HA (D) were compared within the active (viral DNA load > 2000 IU/mL) or inactive (viral DNA load < 2000 IU/mL) HBV carriers according to the LSM score (LSM > 8.5 or <8.5).

Commonly, the ROC curve in logistic regression is a useful approach to predict whether an observation is true or false [34]. Our results of ROC analysis indicated that TAC possessed a sensitivity of 73.91% and a specificity of 64.52% (*p* < 0.02, Figure 1C) for the detection of severe fibrosis in patients with chronic HBV infection; moreover, the APRI also showed a sensitivity of 56.52% and a specificity of 77.42% (*p* < 0.08, Figure 1F). Thus, the combination of the APRI and TAC can be used as a predictor of liver fibrosis degree in HBV carriers due to the simple, rapid, economic and relatively accurate characteristics; moreover, TAC had a higher sensitivity (true positive rate) and more significance for estimating liver fibrosis than the APRI. While the APRI possessed a relatively higher specificity (true negative rate) than TAC, the APRI and TAC might be recommended for simultaneous use in a clinic.

In general, most previous studies have reported that serum ROS, lipid peroxidation, SOD, GSH, and catalase were markedly changed in HBV carriers as compared with healthy controls [21,35]; however, few studies have compared HBV carriers [36]. No notable difference was observed in oxidative stress-related markers between the mild and moderate/severe liver fibrosis groups (Table 2), but we found a significant change in TAC levels in the present study. Actually, the measurement of TAC is merely a reductionist method for evaluating the scavenging capacity of free radicals and predicting holistic antioxidant capacity in vivo [37], which is different from the determination of endogenous antioxidant components, such as SOD, GSH, and catalase [38]. Generally, albumin and uric acid dominantly exert free radical scavenging properties in human serum more than other intraserous components, such as ascorbic acid and α-tocopherol [39]. Clinical studies have reported lower serum albumin levels in liver cirrhosis and a negative correlation between hyperuricemia and liver disease severity in subjects with chronic viral hepatitis [40,41]. In our data, both the serum albumin and uric acid levels were within the normal range, and no difference was observed between the two groups (Table 2). These results indicated the noninvolvement of serum albumin and uric acid in the decreased TAC level in liver fibrosis progression. However, the quality-related aspect of serum albumin would affect the lower TAC results in the moderate/severe fibrosis group. One study reported that the heterogeneity of albumin showed distinct redox potential in vitro [42]. We also found no significant difference in FBG levels between the mild and moderate/severe liver fibrosis groups (Table 2).

**Figure 2:**
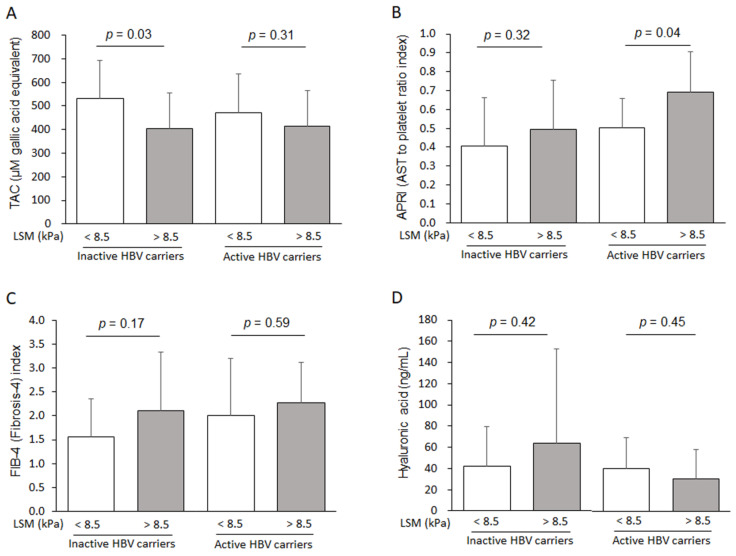
Comparison of the differences in fibrotic markers according to the level of HBV DNA load. The levels of TAC (**A**), APRI (**B**), FIB-4 (**C**), and Hyaluronic acid (**D**) were compared within the active (viral DNA load > 2000 IU/mL) or inactive (viral DNA load < 2000 IU/mL) HBV carriers according to the Liver Stiffness Measurement (LSM) score (LSM < 8.5 or > 8.5).

In addition, we also compared the TAC and APRI levels according to the ”active” versus ”inactive” HBV carriers, which were divided by an HBV viral DNA load of 2000 IU/mL [43]. In general, the pathological features of HBV-derived chronic inflammation and hepatofibrosis are distinguished by the HBV DNA viral load, likely activation of DNA amplification, and the severity of liver injury [44]. Interestingly, TAC was more considerably altered according to the degree of liver fibrosis in inactive HBV carriers (34 subjects, *p* = 0.03), while the APRI (but not FIB and HA) value was more prominent in the active HBV carrier group (20 subjects, *p* = 0.04, Figure 2A–D). This result may indicate that antioxidant capacity may affect the fibrotic change in a more dominant manner in inactive HBV carriers than in active HBV carriers. Regarding the impact of the APRI as an indicator of hepatofibrotic progression, the decrease in platelet number and the increase in AST level in active HBV carriers are well known [45]. Although no significant difference was found in the level of serum transaminases between the mild and moderate/severe liver fibrosis group (LSM > 8.5 versus LSM < 8.5, AST 29.2 ± 10.6 U/L versus 32.3 ± 14.4 U/L, *p* = 0.40, ALT 31.3 ± 15.4 U/L versus 35.4 ± 31.1 U/L, *p* = 0.57, Table 2), our data showed an apparent high level of AST (but not platelet number) in active HBV carriers; furthermore, its level was increased according to the progression of hepatic fibrosis (37.9 ± 13.5 IU/L versus 26.5 ± 9.5 IU/L, *p* < 0.01, Table S2). In addition, we analyzed the possible involvement of antiviral drugs (use or not), even though there were conflicting reports on the prooxidant and antioxidant properties of antiviral agents according to studies [21,46]. Moreover, we also found that the TAC level was notably lower in the moderate/severe fibrotic group (LSM > 8.5 versus LSM < 8.5, 424 ± 136 μM/mL versus 524 ± 166 μM/mL, *p* = 0.06, Table S3) among the antiviral drug use subjects (35 subjects), while the no-antiviral drug use subjects (19 subjects) also displayed an identical patten (LSM > 8.5 versus LSM < 8.5, 390 ± 154 μM/mL versus 486 ± 88 μM/mL, *p* = 0.11, Table S4).

Additionally, TAC and other oxidative stress/antioxidative parameters were compared according to gender difference. However, no significant difference was found in TAC and other oxidative stress/antioxidative parameters between male and female HBV carriers (data not shown). In subgroup analysis, we observed that the TAC levels were significantly lower in the moderate/severe fibrotic group (LSM > 8.5 versus LSM < 8.5, 403 ± 150 μM/mL versus 519 ± 159 μM/mL, *p* = 0.02, Table S5) among the male subjects (44 subjects), while the female subjects (10 subjects) only showed a similar pattern, without statistical significance (LSM > 8.5 versus LSM < 8.5, 461 ± 17 μM/mL versus 516 ± 79 μM/mL, *p* = 0.13, Table S6). A probable explanation is due to the small number of female samples. Although the significant difference in TAC level between the mild and moderate/severe liver fibrosis groups was not detected in female subjects, we cannot simply conclude that gender difference is a crucial factor in the causes of TAC change. Hence, a large sample size and balanced sex ratio should be taken into consideration in further studies.

## 4. Conclusions

Taken together, we can conclude that TAC might become a valuable indicator of the progression of liver fibrosis in HBV carriers, at least in males. As a simple alternative predictor, TAC can assist us to evaluate the extent of liver fibrosis in patients with chronic HBV, along with LSM and biopsies. A relatively small number of samples, especially female and young subjects, is a limitation of this pilot study. Further studies are needed using a large number of samples with evenly distributed age/gender and other causative conditions, such as hepatitis C and alcoholic/nonalcoholic chronic liver diseases.

## Figures and Tables

**Table 1:** Characteristics of participants with hepatitis B virus (HBV).

Characteristics	Male	Female	Total
Subject number	44	10	54
Median age (year, range)	51 (36–66)	54 (45–65)	52 (36–66)
Average height (cm, range)	170 (158–180)	159 (155–174)	168 (155–180)
Average weight (kg, range)	69.5 (49.6–87.0)	57.5 (43.0–70.9)	67 (43.0–87.0)
Mean BMI (value, range)	24.0 (18.4–29.4)	22.8 (18.1–27.2)	23.8 (18.1–29.4)
Antiviral drug +/−	27/17	8/2	35/19
HBV DNA viral load (IU/mL, n)	<2000, 29; >2000, 15	<2000, 6; >2000, 4	<2000, 34; >2000, 20
**Blood/Serum parameters**			
Average AST (U/L)	31 (15–83)	30 (19–60)	31 (15–83)
Average ALT (U/L)	34 (16–166)	29 (14–68)	33 (14–166)
Average GGT (U/L)	37 (11–208)	18 (8–37)	34 (8–208)
Mean Platelets (10^10^/L)	18.1 (8.0–36.4)	13.0 (8.3–19.1)	17.2 (8.0–36.4)
Average FBG (mg/dL)	103 (81–149)	100 (72–122)	103 (72–149)
**Liver fibrotic biomarkers**			
Average LSM (kPa, range)	8.6 (5.5–15.5)	7.7 (5.6–10.1)	8.4 (5.5–15.5)
Mean APRI (value, range)	0.46 (0.15–1.0)	0.64 (0.28–1.44)	0.49 (0.15–1.44)
Mean FIB-4 (score, range)	1.71 (0.74–5.2)	2.65 (1.16–4.69)	1.89 (0.74–5.2)
Mean HA (ng/mL, range)	44 (2–338)	51 (4–145)	45 (2–338)

BMI, body mass index; AST, alkaline aspartate transaminase; ALT, alanine transaminase; GGT, gamma glutamyl transpeptidase; FBG, fasting blood glucose; LSM, liver stiffness measurement; APRI, AST to platelet ratio index; FIB-4, fibrosis-4; HA, hyaluronic acid.

**Table 2:** Parameter changes according to the liver stiffness measurement (LSM) score.

Measurements	Mild Hepatic Fibrosis			Moderate/Severe Hepatic Fibrosis			*t*-Test
	LSM < 8.5 kPa			LSM > 8.5 kPa			
	Total 31 (M/F:23/8)			Total 23 (M/F:21/2)			*p* Value
	Mean		SD	Mean		SD	
LSM (kPa)	6.7	±	0.9	10.8	±	1.8	0.00
TAC (μM/mL)	511.5	±	144.4	406.8	±	143.5	0.01
MDA (μM/mL)	77.5	±	40.2	79.4	±	52.7	0.89
ROS (U/mL)	23.7	±	5.1	23.4	±	6.6	0.84
SOD (U/mL)	6.1	±	3.4	6.0	±	2.7	0.90
Catalase (U/mL)	5.9	±	2.7	4.9	±	2.1	0.13
GSH (μM/mL)	2.4	±	1.3	2.1	±	0.9	0.29
GPx (U/mL)	100.8	±	50.9	88.8	±	33.0	0.30
GRed (U/mL)	49.8	±	17.2	50.1	±	25.8	0.96
APRI	0.4	±	0.2	0.6	±	0.3	0.03
FIB-4	1.7	±	0.9	2.2	±	1.1	0.09
HA (ng/mL)	41.9	±	34.5	49.6	±	70.2	0.63
TGF-β1 (ng/mL)	1.3	±	1.1	1.5	±	0.9	0.47
PDGF (ng/mL)	0.6	±	0.5	0.5	±	0.4	0.71
Platelets (10^10^/L)	18.8	±	6.6	14.9	±	4.5	0.01
Albumin (g/dL)	4.47	±	0.22	4.54	±	0.18	0.20
Uric acid (mg/dL)	2.85	±	0.89	3.03	±	0.93	0.47
AST (U/L)	29.2	±	10.6	32.3	±	14.4	0.40
ALT (U/L)	31.3	±	15.4	35.4	±	31.1	0.57
FBG (mg/dL)	101.2	±	13.1	104.5	±	17.0	0.44

LSM, liver stiffness measurement; TAC, total antioxidative capacity; MDA, malondialdehyde; ROS, reactive oxygen species; GSH, glutathione; GPx, glutathione peroxidase; GRed, glutathione reductase; HA, hyaluronic acid; APRI, AST to platelet ratio index; FIB-4, fibrosis-4; TGF-β1, transforming growth factor-β1; PDGF, platelet-derived growth factor; AST, alkaline aspartate transaminase; ALT, alanine transaminase; FBG, fasting blood glucose.

## Data Availability

The data that support the findings of this study are available from the corresponding author, [Son, C.-G], upon reasonable request.

## Supplementary Materials

The following are available online at https://www.mdpi.com/2076-3921/10/1/77/s1, Table S1: Inclusion criteria and exclusion criteria, Table S2: Parameters change according to the HBV viral DNA load, Table S3: Parameters change in the antiviral drug use subjects according to the LSM score, Table S4: Parameters change in the no-antiviral drug use subjects according to the LSM score, Table S5: Liver fibrosis parameters change in male HBV carriers according to the LSM score, Table S6: Liver fibrosis parameters change in female HBV carriers according to the LSM score.
