# Supplementary material for: Total Antioxidant Capacity in HBV Carriers, a Promising Biomarker for Evaluating Hepatic Fibrosis: A Pilot Study
Source: Antioxidants (Basel). 2021 Jan 8;10(1):77. doi: 10.3390/antiox10010077 (PMC7826661; doi:10.3390/antiox10010077)
Supplement: Supplementary file 1 [file antioxidants-10-00077-s001.docx]

Supplementary tables

| Table S1 Inclusion criteria and exclusion criteria |
| --- |
| **Inclusion criteria** |
| - Participants who are 18 - 65 years old - Participants with chronic liver disorder caused by HBV viral infection - Participants who underwent liver fibrosis with 5.5 kPa to 16 kPa of LSM value using Fibroscan - Participants who are mentally healthy to follow the protocol properly. - Participants who can give informed consent to participate in this trial |
| **Exclusion criteria** |
| - Participants who take immunosuppressive drugs, cytotoxic or hormone therapy - Participants with a history of ascites, variceal bleeding, or hepatic encephalopathy - Participants with marked liver cirrhosis on ultrasonography or CT scan - Participants diagnosed with esophageal varices on endoscopy - Participants with jaundice (Total bilirubin > 3mg/dl) or bleeding tendency (INR > 2.0) - Participants whose AST or ALT is more than 5 times of ULN - Participants with low hemoglobin (< 10g/dl), low number of platelet (< 80,000/mm3), or high serum creatinine (> 1.2-fold of ULN) - Participants who use currently alcohol or drugs - Participants who have severe disorders of heart, lung kidney, blood, gall-bladder, or allergy - Participants with a history of cancer - Woman who are in period of pregnancy or lactation, or planning to have a baby - Participants whose BMI is more than 30 - Participants who cannot understand and cannot follow this trial - Participants who take ursodesoxycholic acid, silymarin, biphenyl dimethyl dicarboxylate, malotilate, colchicine, prophylthiouracil (PTU), or anti-inflammatory agent |
| HBV: Hepatitis B, HCV: Hepatitis C, LSM: liver stiffness measurement, ULN: upper limit of normal, BMI: body mass index |

| Table S2 Parameters change according to the HBV viral DNA load | | | | | | | |
| --- | --- | --- | --- | --- | --- | --- | --- |
| Measurements | HBV viral DNA load (< 2000 IU/mL) | | | HBV viral DNA load (> 2000 IU/mL) | | | T-test |
|  | Total 35 (M/F:29/6) | | | Total 19 (M/F:15/4) | | | *p* value |
|  | Mean |  | SD | Mean |  | SD |  |
| TAC (μM/mL) | 484.0 | ± | 166.5 | 435.4 | ± | 118.3 | 0.22 |
| MDA (μM/mL) | 73.0 | ± | 34.4 | 88.0 | ± | 60.9 | 0.33 |
| ROS (U/mL) | 22.9 | ± | 5.6 | 24.9 | ± | 6.0 | 0.24 |
| SOD (U/mL) | 6.1 | ± | 3.2 | 5.9 | ± | 2.9 | 0.76 |
| Catalase (U/mL) | 5.6 | ± | 2.3 | 5.3 | ± | 3.0 | 0.65 |
| GSH (μM/mL) | 2.3 | ± | 1.0 | 2.1 | ± | 1.3 | 0.66 |
| GPx (U/mL) | 90.8 | ± | 44.7 | 104.6 | ± | 43.0 | 0.27 |
| GRed (U/mL) | 53.1 | ± | 23.5 | 44.2 | ± | 14.5 | 0.09 |
| LSM (kPa) | 8.3 | ± | 2.5 | 8.6 | ± | 2.5 | 0.68 |
| APRI | 0.4 | ± | 0.3 | 0.6 | ± | 0.2 | 0.02 |
| FIB-4 | 1.8 | ± | 1.0 | 2.1 | ± | 1.0 | 0.21 |
| HA (ng/mL) | 50.3 | ± | 61.3 | 35.7 | ± | 28.3 | 0.24 |
| TGF-β1 (ng/mL) | 1.4 | ± | 0.9 | 1.3 | ± | 1.2 | 0.66 |
| PDGF (ng/mL) | 0.6 | ± | 0.6 | 0.3 | ± | 0.3 | 0.01 |
| Platelets (10^10^/L) | 17.1 | ± | 6.0 | 17.3 | ± | 6.4 | 0.90 |
| Monocytes (10^8^/L) | 6.1 | ± | 2.4 | 3.5 | ± | 2.2 | 0.00 |
| AST (IU/L) | 26.5 | ± | 9.5 | 37.9 | ± | 13.5 | 0.00 |
| ALT (IU/L) | 28.2 | ± | 14.6 | 41.9 | ± | 32.5 | 0.09 |
| LSM: Liver Stiffness Measurement; TAC: Total Antioxidative Capacity; MDA: Malondialdehyde; ROS: Reactive Oxygen Species; GSH: Glutathione; GPx: Glutathione Peroxidase; GRed: Glutathione Reductase; HA: Hyaluronic Acid; APRI: AST to Platelet Ratio Index; FIB-4: Fibrosis-4; TGF-β1: Transforming Growth Factor-β1; PDGF: Platelet-Derived Growth Factor | | | | | | | |

| Table S3 Parameters change in the antiviral drug use subjects according to the LSM score | | | | | | | |
| --- | --- | --- | --- | --- | --- | --- | --- |
| Measurements | LSM < 8.5 kPa | | | LSM > 8.5 kPa | | | T-test |
|  | Total 21 (M/F:16/5) | | | Total 14 (M/F:11/3) | | | *p* value |
|  | Mean |  | SD | Mean |  | SD |  |
| TAC (μM/mL) | 523.7 | ± | 166.31 | 424.00 | ± | 135.52 | 0.06 |
| MDA (μM/mL) | 77.6 | ± | 44.49 | 62.03 | ± | 36.97 | 0.27 |
| ROS (U/mL) | 22.9 | ± | 5.04 | 23.90 | ± | 6.63 | 0.63 |
| SOD (U/mL) | 6.6 | ± | 3.93 | 6.11 | ± | 2.49 | 0.66 |
| Catalase (U/mL) | 5.8 | ± | 2.73 | 5.62 | ± | 2.14 | 0.82 |
| GSH (μM/mL) | 2.0 | ± | 0.88 | 2.11 | ± | 0.91 | 0.78 |
| GPx (U/mL) | 89.8 | ± | 45.91 | 77.71 | ± | 32.27 | 0.37 |
| GRed (U/mL) | 52.3 | ± | 19.40 | 50.80 | ± | 30.67 | 0.87 |
| LSM (kPa) | 6.8 | ± | 0.92 | 10.75 | ± | 2.01 | 0.00 |
| APRI | 39.0 | ± | 30.17 | 72.36 | ± | 87.24 | 0.19 |
| FIB-4 | 0.4 | ± | 0.26 | 0.50 | ± | 0.23 | 0.35 |
| HA (ng/mL) | 1.4 | ± | 1.21 | 1.48 | ± | 0.94 | 0.79 |
| TGF-β1 (ng/mL) | 0.6 | ± | 0.61 | 0.54 | ± | 0.53 | 0.60 |
| PDGF (ng/mL) | 18.5 | ± | 5.09 | 13.79 | ± | 3.41 | 0.00 |
| Platelets (10^10^/L) | 523.7 | ± | 166.31 | 424.00 | ± | 135.52 | 0.06 |
| LSM: Liver Stiffness Measurement; TAC: Total Antioxidative Capacity; MDA: Malondialdehyde; ROS: Reactive Oxygen Species; GSH: Glutathione; GPx: Glutathione Peroxidase; GRed: Glutathione Reductase; HA: Hyaluronic Acid; APRI: AST to Platelet Ratio Index; FIB-4: Fibrosis-4; TGF-β1: Transforming Growth Factor-β1; PDGF: Platelet-Derived Growth Factor | | | | | | | |

| Table S4 Parameters change in the no-antiviral drug use subjects according to the LSM score | | | | | | | |
| --- | --- | --- | --- | --- | --- | --- | --- |
| Measurements | LSM < 8.5 kPa | | | LSM > 8.5 kPa | | | T-test |
|  | Total 9 (M/F:8/1) | | | Total 10 (M/F:10/0) | | | *p* value |
|  | Mean |  | SD | Mean |  | SD |  |
| TAC (μM/mL) | 486.3 | ± | 88.5 | 390.1 | ± | 153.9 | 0.11 |
| MDA (μM/mL) | 76.3 | ± | 33.3 | 104.3 | ± | 60.6 | 0.23 |
| ROS (U/mL) | 25.6 | ± | 5.4 | 22.8 | ± | 6.5 | 0.32 |
| SOD (U/mL) | 5.0 | ± | 1.7 | 5.6 | ± | 2.9 | 0.55 |
| Catalase (U/mL) | 6.2 | ± | 3.1 | 4.1 | ± | 1.9 | 0.10 |
| GSH (μM/mL) | 3.2 | ± | 1.7 | 2.1 | ± | 1.0 | 0.13 |
| GPx (U/mL) | 129.2 | ± | 56.0 | 102.9 | ± | 27.7 | 0.23 |
| GRed (U/mL) | 46.4 | ± | 9.2 | 46.9 | ± | 17.6 | 0.94 |
| LSM (kPa) | 6.3 | ± | 0.8 | 10.6 | ± | 1.8 | 0.00 |
| APRI | 37.2 | ± | 28.6 | 27.2 | ± | 26.6 | 0.44 |
| FIB-4 | 0.4 | ± | 0.2 | 0.7 | ± | 0.2 | 0.04 |
| HA (ng/mL) | 1.1 | ± | 0.9 | 1.4 | ± | 1.0 | 0.62 |
| TGF-β1 (ng/mL) | 0.4 | ± | 0.3 | 0.4 | ± | 0.2 | 0.96 |
| PDGF (ng/mL) | 20.4 | ± | 9.3 | 16.1 | ± | 5.7 | 0.24 |
| Platelets (10^10^/L) | 486.3 | ± | 88.5 | 390.1 | ± | 153.9 | 0.11 |
| LSM: Liver Stiffness Measurement; TAC: Total Antioxidative Capacity; MDA: Malondialdehyde; ROS: Reactive Oxygen Species; GSH: Glutathione; GPx: Glutathione Peroxidase; GRed: Glutathione Reductase; HA: Hyaluronic Acid; APRI: AST to Platelet Ratio Index; FIB-4: Fibrosis-4; TGF-β1: Transforming Growth Factor-β1; PDGF: Platelet-Derived Growth Factor | | | | | | | |

| Table S5 Liver fibrosis parameters change according to the LSM score in male HBV carriers | | | | | | | |
| --- | --- | --- | --- | --- | --- | --- | --- |
| Measurements | LSM < 8.5 kPa | | | LSM > 8.5 kPa | | | T-test |
|  | Male: 23 | | | Male: 21 | | | *p* value |
|  | Mean |  | SD | Mean |  | SD |  |
| TAC (μM/mL) | 518.8 | ± | 159.3 | 402.5 | ± | 149.8 | 0.02 |
| LSM (kPa) | 6.6 | ± | 0.9 | 10.9 | ± | 1.9 | 0.00 |
| APRI | 0.4 | ± | 0.1 | 0.6 | ± | 0.3 | 0.00 |
| FIB-4 | 1.4 | ± | 0.4 | 2.1 | ± | 1.1 | 0.01 |
| HA (ng/mL) | 36.9 | ± | 29.7 | 51.7 | ± | 73.2 | 0.39 |
| LSM: Liver Stiffness Measurement; TAC: Total Antioxidative Capacity; HA: Hyaluronic Acid; APRI: AST to Platelet Ratio Index; FIB-4: Fibrosis-4 | | | | | | | |

| Table S6 Liver fibrosis parameters change according to the LSM score in female HBV carriers | | | | | | | |
| --- | --- | --- | --- | --- | --- | --- | --- |
| Measurements | LSM < 8.5 kPa | | | LSM > 8.5 kPa | | | T-test |
|  | Female: 7 | | | Female: 3 | | | *p* value |
|  | Mean |  | SD | Mean |  | SD |  |
| TAC (μM/mL) | 516.0 | ± | 79.0 | 461.3 | ± | 17.1 | 0.13 |
| LSM (kPa) | 7.0 | ± | 0.7 | 9.4 | ± | 1.1 | 0.05 |
| APRI | 0.6 | ± | 0.4 | 0.6 | ± | 0.2 | 0.90 |
| FIB-4 | 2.6 | ± | 1.5 | 2.7 | ± | 0.7 | 0.92 |
| HA (ng/mL) | 43.9 | ± | 29.3 | 66.6 | ± | 69.1 | 0.63 |
| LSM: Liver Stiffness Measurement; TAC: Total Antioxidative Capacity; HA: Hyaluronic Acid; APRI: AST to Platelet Ratio Index; FIB-4: Fibrosis-4 | | | | | | | |
